# Comparative evaluation of antibacterial effect of nanoparticles and lasers against Endodontic Microbiota: An in vitro study

**DOI:** 10.4317/jced.55076

**Published:** 2018-12-01

**Authors:** Vibha Kushwaha, Rakesh-Kumar Yadav, Aseem-Prakash Tikku, Anil Chandra, Promila Verma, Prashant Gupta, Vijay-Kumar Shakya

**Affiliations:** 1 Junior Resident, Department of Conservative Dentistry and Endodontics, King George’s Medical University, Lucknow, U.P., India; 2 MDS, Professor (Junior Grade), Department of Conservative Dentistry and Endodontics, King George’s Medical University, Lucknow, U.P., India; 3 MDS, Professor and Head, Department of Conservative Dentistry & Endodontics, Faculty of Dental Sciences, King George’s Medical University, Lucknow, U.P., India; 4 MDS, Professor, Department of Conservative Dentistry and Endodontics, King George’s Medical University, Lucknow, U.P., India; 5 MDS, Associate Professor, Department of Microbiology, King George’s Medical University, Lucknow, U.P., India; 6 MDS, Assistant Professor, Department of Conservative Dentistry and Endodontics, King George’s Medical University, Lucknow, U.P., India

## Abstract

**Background:**

Present study was conducted with the aim of evaluating antimicrobial efficacy of silver (AgNP) and gold nanoparticles (AuNP) with and without Nd: YAG laser (L) irradiation against experimentally inoculated *Enterococcus faecalis* in infected human root dentin.

**Material and Methods:**

120 extracted single rooted human teeth were prepared and inoculated with *E. faecalis* for 24 hrs. The teeth were then randomly divided into 4 experimental group: AgNPs group: irrigation for 3minutes with 50 μl of 100 ppm, the AuNPs group: irrigation with 50 μl of 100 ppm, the AgNPs & Nd: YAG lasers group: irrigation with 50 μl of 100 ppm + irradiation with 1.5W laser for 60 seconds, the AuNPs & Nd: YAG lasers group: irrigation with 50 μl of 100 ppm + irradiation with 1.5W laser for 60 seconds. One control group consisting of 2% CHX irrigation for 3 minutes was also there (n = 20). The specimens were collected from the canal before and after irrigation, and colony forming units were observed.

**Results:**

Significant difference was found among all the groups in comparison to the control group (*p*<0.05). The greatest reduction in CFU’s was observed with combination of AgNPs & Nd: YAG lasers group.

**Conclusions:**

AgNPs in combination with Nd: YAG laser irradiation has the potential to be used as root canal disinfectant.

** Key words:**Antibacterial efficacy, gold, silver, nanoparticles, lasers, solid-state.

## Introduction

Polymicrobial infection encountered inside the root canal system is one of the major challenge that is faced by an endodontists (1,2). Although variety of microbes are cultured from the infection of root canal system depending upon the type of infection e.g. primary endodontic infection is seen colonized mainly by Bacteroides, Porphyromonas, Prevotella, Fusobacterium, Treponema, etc, whereas in secondary infection, enterococcus, Actinomyces, streptococcus and candida species are seen (2). *Enterococcus faecalis* is a gram positive cocci, which is reported to be present in approximately 20-30% of primary infections and 67-77% of secondary infections (3). It has a unique morphological and genetic composition, which enables it to form one of the most resistant biofilm that can resist the action of various antimicrobials used inside the root canal (4). Various virulence factors have been isolated from *E. faecalis* such as collagen binding proteins, aggregation substances, gelatinase etc. all of which account for greater resistance and a very strong defense mechanism (5).

Mechanical cleaning in the form of cleaning and shaping reduces the bacterial load but not completely (6). Chemical disinfection in the form of irrigation and Intracanal medicaments are also employed but the resistant microbes in the form of *E. faecalis* are able to overcome their action resulting in the persistent infection (7). 2% Chlorhexidine (CHX) has been shown to be the most efficacious medicament against *E. faecalis* due to its bisguanide nature that interacts with the negatively charged bacterial surface and causes cellular disintegration (8,9). But owing to the complexity of root canal system in the form of lateral canals, isthmuses and various other ramifications, irrigants are not able to reach these critical zones leading to the persistence of bacteria and secondary failure later on (10,11).

Nanoparticles are very small sized particles that have greater surface area and exert their antimicrobial effect by interacting with the negatively charged bacterial cell wall. Various nano particles have been tested against the microbes for their antimicrobial efficacy e.g. silver (Ag), gold (Au), Zinc (Zn) and out of these silver nanoparticles have been found to be the most effective (12). Not much of literature is available that has evaluated the effect of gold nanoparticles against *E. faecalis*.

Lasers have become increasingly popular in dentistry due to their multiple uses and one such important application is disinfection of the root canal system. Evidence suggests that lasers can penetrate to depths grater then 400μ inside the dentinal tubules and cause the killing of bacteria in these greater depths (13). Evidence suggests the bactericidal effect of Nd: YAG laser against *E. faecalis* when used in various activation modes at different settings (14,15). Lasers apart from causing bactericidal effect also cause occlusion of the dentinal tubules and thereby closing all the avenues of bacterial reinfection.

Very limited data is available as far as the evaluation of combined effect of nanoparticles irrigation and Nd: YAG laser with respect to their action against *E. faecalis* is concerned. Although in a study by Afkhami *et al.* combined effect of silver nanoparticles and diode laser and photodynamic therapy was evaluated and promising results were obtained by the authors (16). But the effects of nanoparticles irrigation and Nd: YAG laser irradiation has not been evaluated and the present study was conducted with the aim of evaluating the antimicrobial effect of silver and gold nanoparticles in combination with Nd: YAG laser irradiation against *E. faecalis* in *In vitro* conditions. The null hypothesis was that there will be no difference in the antimicrobial efficacy of both the nanoparticles in combination with Nd: YAG laser irradiation against *E. faecalis*.

## Material and Methods

-Preparation of teeth.

Present study was conducted after obtaining ethical clearance from the institutional ethics committee (Ref.code 84th ECM II B- Thesis / P3). 120 freshly extracted single rooted human teeth, extracted for the orthodontic purpose were obtained and written consent was taken for every patient. Only those teeth that radiographically confirmed to have single canal were included in the study. Roots were decoronated at a distance of 14mm from the apex and stored in 0.2% thymol solution until further use. Working length short of 1mm from the apex was used the reference point for cleaning and shaping of canals. Cleaning and shaping was performed using Protaper universal files (Dentsply Maillefer, Ballaigues, Switzerland) up to F4, which was the master apical file. All instrumentation was done using GlydeTM (Dentsply Maillefer, Ballaigues, Switzerland) as a lubricating agent for assisting the motion of file inside the canal. In between the instrumentation 3ml of 3% NaOCl was used as irrigating solution. At the end of the mechanical preparation 3ml of 3% NaOCl followed by 3ml of Ethylene diamine tetra acetic acid (EDTA) solution was used to remove the smear layer. Final irrigation was performed using physiological saline solution. Two layers of nail polish was applied on the outer tooth surface and apex was sealed with self-cure glass ionomer cement. The teeth were then transferred into 2ml micro tubes and autoclaved at 121°C under 15 lbs pressure for 15 min.

-Microbiological procedures:

Clinical isolates of *E. faecalis* were used as the test microorganisms. Bacterial colonies isolated for 24 h were suspended in 5ml of MacConky agar without crystal violet and then incubated at 37°C for 4 h.

The 120 root specimens were transferred into sterile cell culture well plates (each plate having 24 wells) under sterile conditions. Roots were mounted in the well plates containing 2% sterile agar media which was allowed to solidify so that root specimens can be stabilized. After stabilization of teeth specimen on cell culture plates, 50μl of *E. faecalis* was injected in the respected root specimens using micropipette with sterile needle. After injection tubes were incubated anaerobically at 37°C for 4 weeks to form a biofilm. Every alternate day 10μ of fresh broth was added to ensure the viability of bacteria.

At the end of 4 weeks a baseline sample was obtained from the specimens before canal disinfection. For baseline sampling, root specimens were flushed with normal saline solution using 30 gauge needle and dentin was scrapped using #35Hedström file (Dentsply M access, Ballaigues, Switzerland). A #35 sterile paper point (Dentsply, Ballaigues, Switzerland) was placed inside the canals for 60 seconds and was then transferred into a sterile microtube containing 1 mL saline solution and vortexed for 30 seconds. A serial dilution up to 1:100 was prepared. Then 0.1ml aliquot of each dilution was spread on agar plates containing MacConky agar without crystal violet and then incubated at 37°C for 24 h. After 24hrs the total number of colony forming units were counted and recorded on a Microsoft excel sheet for further evaluation.

-Experimental groups.

The specimens were randomly divided into 2 control group (n = 20) and 4 experimental groups (n = 20):

Group I (Negative control group): root canals were irrigated with 3ml of normal saline using 30 gauge needle attached to a 3ml syringe. The solution was allowed to remain in contact with the canal for 3minutes.

Group II (2% Chlorhexidine (CHX)): root canals were irrigated with 3ml of 2% CHX (Prevest Denpro Limited, Jammu, India) using 30 gauge needle attached to a 3ml syringe. The solution was allowed to remain in contact with the canal for 3minutes.

Group III (Silver nano particles (SNP)): root canals were irrigated with 3ml Of SNP solution Reinste Nano ventures Pvt. Ltd, New Delhi, India) having concentration of 100ppm and average particle size of 20nm, using 30 gauge needle attached to a 3ml syringe. The solution was allowed to remain in contact with the canal for 3minutes and then aspirated using the same needle.

Group IV (Gold nano particles (GNP)): root canals were irrigated with 3ml Of GNP solution (Reinste Nano ventures Pvt. Ltd, New Delhi, India) having concentration of 100ppm and average particle size of 20nm, using 30 gauge needle attached to a 3ml syringe. The solution was allowed to remain in contact with the canal for 3minutes and then aspirated using the same needle.

Group V (SNP + Nd: YAG Laser): root canals were irrigated with 3ml Of SNP solution (Reinste Nano ventures Pvt. Ltd, New Delhi, India) having concentration of 100ppm and average particle size of 20nm, using 30 gauge needle attached to a 3ml syringe followed by simultaneous irradiation of the canal with Nd: YAG laser delivered by 200 um fiber tip kept 1-2 mm away from the apical canal and activated at 15Hz, 100mJ, 2W power setting in sweeping motion from apical to coronal till 60 sec.

Group VI (GNP + Nd: YAG Laser): root canals were irrigated with 3ml Of GNP solution (Reinste Nano ventures Pvt. Ltd, New Delhi, India) having concentration of 100ppm and average particle size of 20nm, using 30 gauge needle attached to a 3ml syringe followed by simultaneous irradiation of the canal with Nd: YAG laser delivered by fiber tip and activated at 15Hz, 100mJ, 2W power setting.

-Incubation and Microbial sampling of Experimental groups:

Each root canal specimen was flushed with 5ml of sterile saline. The specimen medicated with 2% CHX solution were neutralized with 0.5% Tween 80 in 0.07% lecithin, and again irrigated with 5ml sterile saline.

After irrigation with physiological saline solution, the root specimens were dried using # 35 paper point. Samples were obtained by scrapping # 35 Headstrom file inside the root canal and then transferring this file into the sterile micro centrifuge tubes containing 1ml of saline solution and vortexed for 30 seconds. Bacterial culture was prepared as described earlier for the baseline samples.

-Data analysis.

The results are presented in mean±SD. One way analysis of variance followed by Tukey’s post-hoc tests was used to compare CFU among the groups. The *p*-value<0.05 was considered significant. All the analysis was carried out on SPSS 16.0 version (Chicago, Inc., USA).

## Results

**Figure 1:**
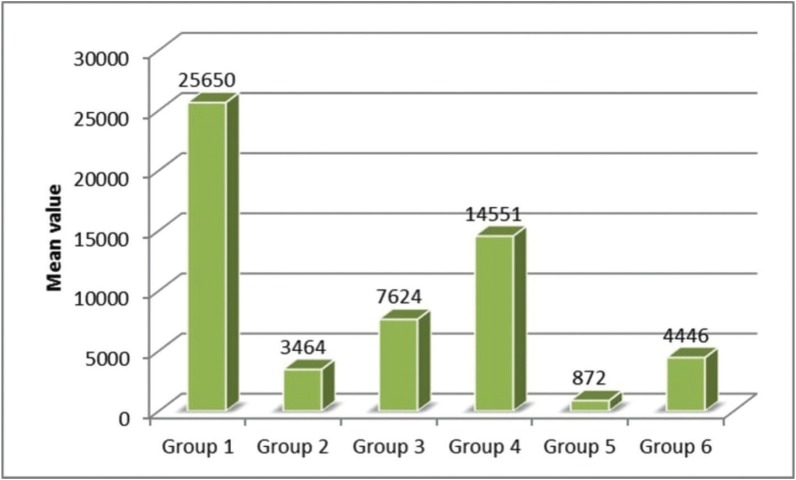
Comparison of CFU among the groups.

Analysis of variance showed that there was significant (*p*=0.0001) difference in CFU among the groups (*p*<0.05).  Table 1 and figure 1 show the inter group comparison on the basis of colony forming units. The lowest CFU were noted in Group V (SNP + Nd: YAG Laser) and highest CFU were noted in Group IV (Gold nano particles (GNP)) among experimental groups. Intergroup comparison between Group V (SNP + Nd: YAG Laser) and Group II (2% CHX) was also found to be significant. Significant differences was observed between Group III and IV i.e. (SNP) and (GNP) group. Although significant differences were noted among all the groups (*p*<0.05) but inter group comparison between group II and group VI was found to be insignificant (*p*>0.05).

**Table 1:**
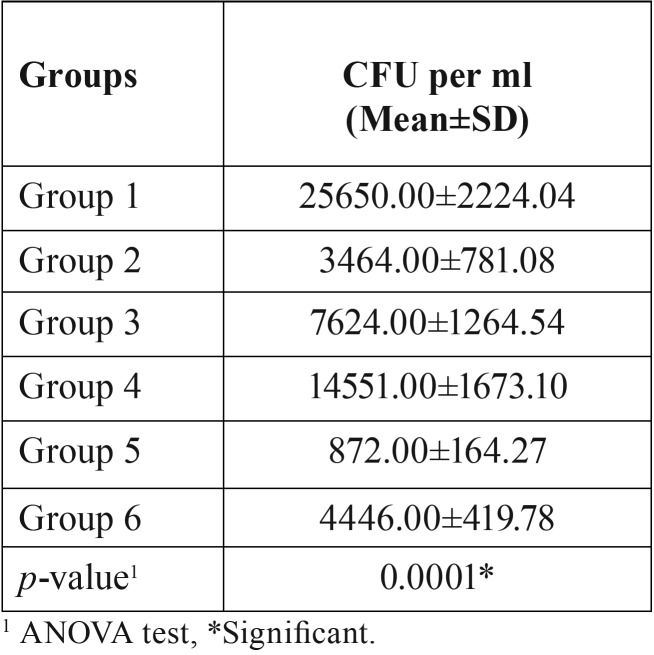
Comparison of CFU among the groups.

## Discussion

Complexity of the root canal system, polymicrobial nature of infection and tendency of the microbes to form biofilm are the major obstacle in predictable disinfection of root canal system (1,7). Biofilms formed by the gram positive *E. faecalis*, which is encountered in most of the secondary infection cases are the most resistant biofilm that overcome the antimicrobial action of the routinely used irrigants and intracanal medicaments such as sodium hypochlorite and calcium hydroxide (4). Hence in the present study *E. faecalis* was used as the test microorganism against which the antimicrobial efficacy was evaluated.

Studies have shown that microbes respond differently to same medication under different conditions i.e. planktonic and biofilm forms (17). So in order to simulate the resistant biofilm conditions, in the present study *E. faecalis* biofilm was formed by incubating the colonies for 4 weeks at 37°C and in order to ensure the viability of the bacteria, fresh broth was added every alternate day so that no bacteria died of starvation.

Studies have shown that nanoparticles and lasers have considerable antimicrobial action against *E. faecalis* (12,14,16,18) and studies have also evaluated the antimicrobial action of silver nanoparticles in combination with photodynamic therapy and have found significant results against *E. faecalis* (16). But we could not found any study that had evaluated the antimicrobial efficacy of silver and gold nanoparticles when used with simultaneous Nd: YAG laser irradiation. Owing to the antimicrobial property of the Nd: YAG lasers against *E. faecalis* and their ability to penetrate to greater depths inside the dentinal tubules, present study was conducted with the aim of evaluating the effect of silver and gold nanoparticles irrigation followed by simultaneous Nd: YAG irradiation in eliminating *E. faecalis* biofilms.

In the present study positive control group (2% CHX), showed significant reduction in Enterococcus colonies in comparison to the negative control group and these findings are in agreement with the results reported earlier where 2% CHX has been found to be highly effective against *E. faecalis* (4,19).Although significant reduction in the microbial colonies have been reported by the studies earlier by using various irrigants but the major limitation is the ability to eliminate the bacteria from within the depths of dentinal tubules. Irrigants have the limitation in penetrating only to the superficial depths of 200μ whereas bacteria’s can get lodged to a depth of 1000μ (20).

Nanoparticles are newer class of antimicrobials that have positively charged high surface area that interact with the negatively charged bacterial cell wall and exert their antimicrobial effect. To the best of our knowledge, antimicrobial efficacy of silver nanoparticles has been reported in the literature by various authors but their combined effect when used with simultaneous Nd: YAG laser irradiation has not been reported and it will be very interesting to know the antimicrobial efficacy of silver and gold nanoparticles when used with simultaneous Nd: YAG laser irradiation as lasers can penetrate to dentinal depths of more than 1000μ (13).

In the current study, simultaneous irradiation of Nd: YAG laser and SNP irrigation (Group V) showed significantly better results in terms of colony forming units in comparison to other groups. Although to the best of our knowledge no other study has evaluated the antimicrobial efficacy using same strategy but in a study by Rahimi *et al.* (21) Significantly better results were reported when NaOCl irrigation was used in combination with Nd: YAG laser irradiation against *E. faecalis*. Results obtained in the current study can be attributed to the deep dentinal tubule penetration by Nd: YAG laser followed by high surface charge characteristics of silver nano particles.

Among various lasers available, Nd: YAG laser is probably one of the most effective laser for disinfection of root canal infected with *E. faecalis* (15). In a study by Bergman’s *et al.* (22) a reduction of 99.97% of *E. faecalis* colonies was found when Nd: YAG laser was used alone. Although in the present study, individual effects of Nd: YAG laser irradiation were not observed rather combination of laser with nanoparticles was evaluated but the significantly better results obtained can be attributed to the deep penetration effects of laser followed by antimicrobial effects of nanoparticles.

In the current study significantly different results were obtained when Nd: YAG laser irradiation was used in combination with silver and gold nanoparticles. We could not find any relevant literature to explain the present result but in a study Hernández-Sierra JF *et al.* (23), higher antimicrobial efficacy of SNP was found against *Streptococcus mutans* in comparison to AU and ZnO nanoparticle. Silver has an important antimicrobial effect. This effect is dependent on superficial contact, in that silver can inhibit enzymatic systems of the respiratory chain and alter DNA synthesis. In many studies reported earlier also gold has shown a weak antimicrobial effect against many microorganisms, and its use in oral pathology is uncommon (24).

Although the present study was conducted with standard protocols and guidelines but even then it has certain limitations. Major limitation of the following study was that it was not entirely an *In vivo* study. Although every possible step was taken to create natural simulating conditions but even then the real dynamism of purely *in vivo* conditions can never be achieved. Secondly, the antimicrobial effect achieved with various groups in the present study were obtained in *In vitro* conditions. Whenever a secondary root canal infection occurs, it’s never mono microbial rather poly microbial in nature. Hence the results might change in the presence of other microbes due to various microbial interactions.

## Conclusions

Application of Nd: YAG laser in combination with silver nanoparticles irrigation had significant effects in the reduction of microbial colonies of *Enterococcus faecalis* in comparison to other experimental groups. No significant differences were found among Au + Nd: YAG laser group and 2% CHX group, although significant difference existed among rest of the groups. Present study highlights the potential of this method in decreasing microbial load inside the root canal. Although promising results were obtained in the present study but the growing concerns of nanoparticles induced cell toxicity and their effects on viable tissues needs to be evaluated prior to their incorporation as routine endodontic irrigant.
